# Analyzing the association between age-related macular degeneration, retinal vascular occlusions, and dementia

**DOI:** 10.1177/11206721261438587

**Published:** 2026-04-07

**Authors:** Ahmed Alshaikhsalama, Krista Thompson, Haafiz Hashim, Kishan G Patel

**Affiliations:** 1 12334 University of Texas Southwestern Medical School, Dallas, TX, USA; 2 Department of Ophthalmology, 12334 University of Texas Southwestern Medical Center, Dallas, TX, USA

**Keywords:** AMD, RAO, RVO, BRVO, CRVO, dementia, vascular dementia, Alzheimer's dementia, all cause dementia

## Abstract

**Purpose:**

To investigate the association between age-related macular degeneration (AMD), retinal artery occlusion (RAO), and retinal vein occlusion (RVO) and the future development of Alzheimer's disease (AD), vascular dementia (VaD), and all-cause dementia (ACD).

**Methods:**

Retrospective propensity score–matched cohort study using TriNetX, a confederated healthcare network. The study population included 91,296 AMD, 15,372 RAO, 35,862 RVO, and 3,076,291 population control (PC) patients aged 65 and older. Propensity score matching was applied to control for baseline demographics and health characteristics. The primary outcome was the measured risk of developing AD, VaD, and ACD following incident diagnosis of AMD, RAO, or RVO.

**Major Findings:**

AMD was associated with a significantly elevated risk of AD (HR, 1.25; 95% CI, 1.17–1.34; *P* < 0.001), VaD (HR, 1.20; 95% CI, 1.11–1.31; *P* < 0.001), and ACD (HR, 1.12; 95% CI, 1.08–1.16; *P* < 0.001) following diagnosis with an average follow up of 45.0 ± 36.7 months. RVO patients also displayed higher risks across all dementia types (AD: HR, 1.46; 95% CI, 1.22–1.74; *P* < 0.001; VaD: HR, 1.51; 95% CI, 1.23–1.87; *P* < 0.001; ACD: HR, 1.34; 95% CI, 1.22–1.47; *P* < 0.001) with an average follow up of 46.6 ± 37.9 months.

**Conclusions:**

Retinal disease diagnoses may correlate with increased dementia risk. While AMD and RVO are associated with all dementias (AD, VaD, and ACD), RAO did not increase dementia risk.

## Introduction

Age-related macular degeneration (AMD), retinal vein occlusion (RVO), and retinal artery occlusion (RAO) are significant causes of vision loss.^1–3^ In addition to ocular morbidity, these retinal diseases have multiple similarities to common causes of dementia, leading prior studies to hypothesize a pathophysiological link.^4,5^

AMD shares many environmental risk factors with Alzheimer's disease (AD), including tobacco use, obesity, atherosclerosis, hypertension, and hypercholesterolemia.^4,6^ Furthermore, biochemical analyses have revealed a major constituent of drusen to be amyloid beta (Aβ) peptide, the primary hallmark of AD.^5^ RAO and RVO also have risk factors associated with dementia, including metabolic syndrome, carotid stenosis, peripheral vascular disease, and history of vascular cerebral stroke.^7^

Given their hypothesized shared pathogenesis, multiple studies have analyzed the epidemiological relationship between RVO, RAO, AMD and dementia, with conflicting results. A 2014 study examining English health service patients did not find any association between hospital admissions for AMD and AD.^8^ However, a meta-analysis summarizing 25 studies and over 11 million participants found an association between AMD and all cause dementia (HR, 1.29; 95% CI, 1.13–1.48) as well as AMD and AD (HR, 1.27; 95% CI, 1.06–1.52), albeit with significant inter-study variation.^9^

The association between retinal vascular occlusions and dementia has also been inconclusive. A 2023 study in Denmark examining over 2 million patients did not find RAO to be a risk factor for VaD after matching for baseline covariates.^10^ However, a 2025 study showed an increased prevalence of AD and ACD in patients with RAO amongst the entire UK Biobank population.^11^ Similarly, data on the relationship between RVO and dementia has been mixed. A study examining over 40,000 RVO patients in South Korea found a 1.16, 1.15, and 1.24 HR for all-cause dementia (ACD), AD, and VaD, respectively.^12^ However, a systematic review and meta-analysis of six cohort studies found RVO to be associated with an increased risk of vascular dementia alone, with no significant increased risk of AD or ACD.^13^ This finding is further supported by systematic reviews by Chen and colleagues demonstrating a strong association between RVO and cerebrovascular accidents, myocardial infarctions, and leukemia.^14–16^ Vascular dementia is widely regarded as a clinical manifestation of substantial vascular disease burden. Accordingly, RVO may serve as both an indicator and a component of this broader systemic vascular pathology.

Given these conflicting results, the purpose of this investigation was to analyze whether a diagnosis of AMD, RVO, and RAO is a risk factor for the future development of AD, VaD, and ACD by analyzing a large, national, collaborative health database.

## Methods

This retrospective study examined four primary patient study cohorts: AMD, RVO, RAO, and a population control (PC) group. All data was collected via the TriNetX network (TriNetX Inc, Cambridge, MA, United States), a database incorporating de-identified data including 135 million patients from 66 health care organizations across the U.S. International Classification of Diseases Tenth Revision (ICD-10) codes were used to categorize and measure diseases in this study; for a glossary of ICD-10 codes used, refer to Supplemental Table 1. Data was collected between August 1, 2004, to August 1, 2024. The STROBE guidelines were followed in this report.^17^

Data for this study was extracted on August 12^th^, 2024. Cohorts were constructed by querying the database using specific inclusion and exclusion criteria. The inclusion criteria for each retinal disease group was a diagnosis of AMD, RVO, or RAO in patients over 65. Each retinal disease cohort (e.g., RAO) excluded a prior or future history of other retinal diagnoses examined in this study (e.g., AMD and RVO) to limit confounding subsequent retinal diagnoses, as well as a history of outcomes (AD, VaD, or ACD) prior to the diagnosis of retinal disease. The PC cohort was created using the ICD-10 code for general adult medical examination without abnormal findings (Z00.00), while excluding any history or future incidence of the examined retinal diseases in this study, along with any prior history of dementia (AD, VaD, or ACD). Patients with less than 1 year of follow up or 1 year of prior medical records were excluded from all cohorts.

The index date for the retinal disease groups was defined as the first instance of a retinal disease diagnosis. The lookback period required at least one year of prior medical records and included up to 20 years of look-back prior to index retinal disease diagnosis to ensure only incident retinal diagnoses were evaluated. The index date for the PC cohort was defined as the first date of medical evaluation where demographic and systemic covariates matched retinal disease cohorts. Patients were censored at the development of any dementia diagnosis, death, loss of follow up, or end of the study period which included up to 10 years of records following index diagnosis.

A post hoc sensitivity analysis was conducted. This analysis aimed to reduce potential bias caused by excluding patients based on future diagnostic transitions, which could have influenced effect estimates. The analysis included the initial RAO, RVO and AMD cohorts over 65 without prior or future exclusion of comparator retinal diagnoses (e.g., A patient with RAO could develop RVO at a later point in the study period) compared with PC controls. Patients with prior diagnoses of outcome measures (dementia diagnoses) or less than one year of follow-up were excluded.

### Statistical analysis

Collected data included baseline demographics, systemic comorbidities, and lab values prior to AMD, RVO, and RAO diagnoses. Examined outcomes included incident AD, VaD and ACD diagnosis following initial AMD, RVO, and RAO diagnoses. Secondary outcomes included development of dementia in subgroups of AMD (exudative and nonexudative AMD), and RVO (branch retinal vein occlusion (BRVO) and central retinal vein occlusion (CRVO)). For the AMD subgroups, nonexudative AMD patients excluded a history or future incidence of exudative AMD, while the exudative AMD cohort excluded patients with a future diagnosis of nonexudative AMD. For the RVO subgroups, a prior or future history of its partner subgroup was excluded e.g., BRVO subgroup excluded any prior or future history of CRVO. RAO subgroups were not examined due to limited sample size.

**Figure 1.**
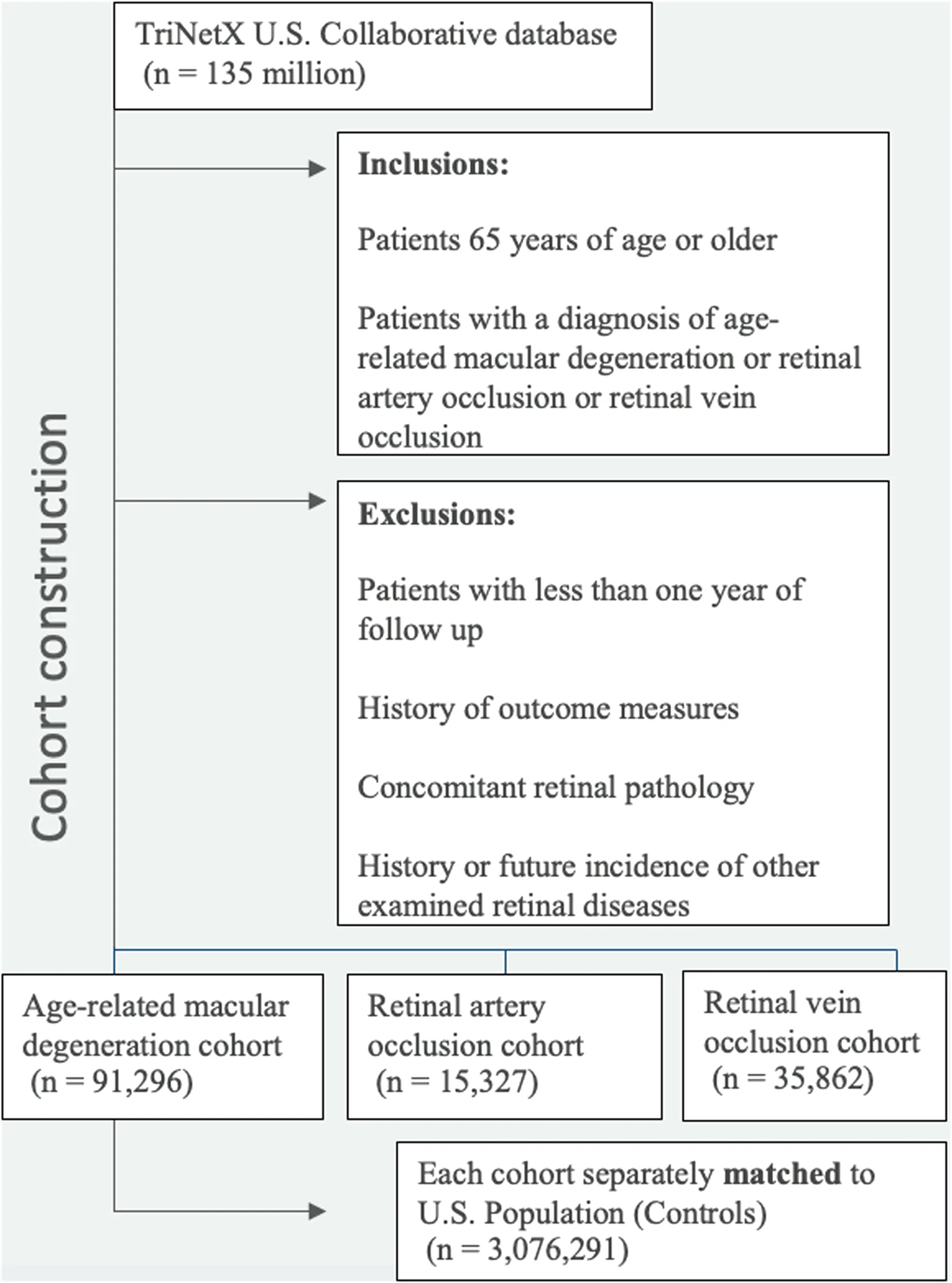
Cohort construction. Cohorts were constructed utilizing the TriNetX collaborative database as outlined above.

Outcomes for each retinal disease cohort were compared to a subset of the PC group using propensity score matching (PSM) to factor for age, sex, race, body mass index (BMI), and systemic comorbidities. PSM consisted of 1:1 nearest-neighbor (greedy) matching without replacement. We used a caliper of 0.10 SD applied on the propensity score scale. Only patients with an available propensity score were eligible for matching; patients with missing data that prevented propensity score estimation were excluded. The standardized mean difference (SMD) was calculated for covariates, with a computed SMD ≤ 0.1 indicating appropriate statistical balance. Figure 1 summarizes cohort development for the retinal disease and PC cohorts. Systemic comorbidities included hypertension (HTN), hyperlipidemia (HLD), ischemic heart disease (IHD), diabetes mellitus (DM) including hemoglobin A1c (HbA1c) level, chronic kidney disease (CKD), cerebrovascular disease (CVD), and nicotine dependence. Data analysis was performed using built-in TriNetX analytics (TriNetX, LLC). After matching, hazard ratios (HRs) with 95% confidence intervals (CIs) were used to compare cohorts for the development of dementia outcomes through 10 years by factoring in time-to-event between cohorts and the number of patients experiencing each dementia diagnosis following AMD, RVO, and RAO diagnoses. The Schoenfeld residual approach was used within the TriNetX platform to test the proportional hazards assumption. Continuous variables were analyzed with independent t tests, and categorical variables with chi-square tests. HR CIs were calculated on the log scale assuming approximately normal distribution. Statistical significance was set at two-sided *P* < 0.05.

## Results

Between 2004 and 2024, 91,296 patients developed AMD, 15,372 patients developed RAO, and 34,862 patients developed RVO. The PC cohort initially consisted of 3,076,291 patients. Before PSM, patients with AMD, RAO, and RVO had significant differences in age, race, gender, and were more likely to have the following pre-existing co-morbidities when compared to the PC cohort: HTN, DM, HLD, IHD, CVD, and CKD. After PSM, no significant differences compared to PC were observed between baseline characteristics across all cohorts as shown in Tables 1a–c.

**Table 1a.** Baseline characteristics of AMD and control cohort before and after propensity score matching.

	Before PSM			After PSM		
	AMD (N = 91,296)	Control (N = 3,076,291)	SMD	AMD (N = 91,296)	Control (N = 91,296)	SMD
Age at index, years (Mean +/- SD)	77.8 ± 8.6	68.5 ± 8.7	1.30	77.8 ± 8.6	77.8 ± 8.6	0.01
*Gender*	* *					* *
Female	54.1%	52.6%	0.10	54.1%	54.2%	0.01
Male	35.8%	41.6%	0.11	35.8%	35.7%	0.01
Unknown Gender	10.1%	5.8%	0.16	10.1%	10.1%	0.00
*Race*						
White	72.7%	71.1%	0.10	64.4%	64.8%	0.01
Black	3.00%	10.7%	0.28	3.0%	3.1%	0.00
Asian	2.4%	2.9%	0.05	2.4%	2.4%	0.00
Unknown Race	21.9%	15.3%	0.10	30.2%	29.7%	0.01
*Systemic comorbidities*						
Hypertension (HTN)	47.7%	33.5%	0.17	47.7%	47.8%	0.01
Diabetes Mellitus	27.7%	12.8%	0.14	27.7%	27.6%	0.00
Hyperlipidemia (HLD)	37.1%	22.4%	0.14	37.1%	37.0%	0.01
Ischemic Heart Diseases	14.7%	6.1%	0.31	14.7%	14.7%	0.00
Chronic Kidney Disease (CKD)	9.0%	3.2%	0.26	9.0%	8.8%	0.00
Nicotine Dependence	12.3%	9.3%	0.00	12.3%	12.2%	0.03
Cerebrovascular Diseases	8.6%	3.2%	0.33	8.6%	8.5%	0.00
*Lab Data*						
BMI (kg/m^2^)	30.0 ± 7.2	29.9 ± 7.0	0.16	30.0 ± 7.2	30.1 ± 6.9	0.05
HbA1c	6.6 ± 2.0	6.3 ± 1.8	0.07	6.6 ± 2.0	6.6 ± 2.0	0.04

PSM: Propensity Score Matching; SD: Standard Deviation; SMD: Standardized mean difference; CKD: Chronic Kidney Disease; BMI: Body Mass Index; HbA1c: Hemoglobin A1c; HTN: Hypertension; HLD: Hyperlipidemia, N: Number of patients.

**Table 1b.** Baseline characteristics of RVO and control cohort before and after propensity score matching.

	Before PSM			After PSM		
	RVO (N = 34,862)	Control (N = 3,076,291)	SMD	RVO (N = 34,862)	Control (N = 34,862)	SMD
Age at index, years (Mean +/- SD)	73.1 ± 9.0	68.5 ± 8.7	0.52	73.1 ± 9.0	73.1 ± 9.0	0.00
*Gender*	* *					* *
Female	46.7%	52.6%	0.12	46.7%	46.4%	0.01
Male	49.6%	41.6%	0.16	49.6%	49.9%	0.01
Unknown Gender	3.7%	5.8%	0.10	3.7%	3.7%	0.00
*Race*						
White	62.6%	71.1%	0.18	57.7%	58.0%	0.01
Black	15.0%	10.7%	0.13	15.0%	15.0%	0.00
Asian	4.0%	2.9%	0.06	4.0%	4.0%	0.00
Unknown Race	18.4%	15.3%	0.08	23.3%	23.0%	0.01
*Systemic comorbidities*						
Hypertension (HTN)	66.0%	33.5%	0.65	66.0%	65.5%	0.01
Diabetes Mellitus	34.2%	12.8%	0.50	34.2%	33.8%	0.01
Hyperlipidemia (HLD)	45.6%	22.4%	0.49	45.5%	45.2%	0.01
Ischemic Heart Diseases	13.6%	6.1%	0.25	13.6%	13.5%	0.00
Chronic Kidney Disease (CKD)	17.3%	3.2%	0.46	17.3%	16.6%	0.02
Nicotine Dependence	9.3%	9.3%	0.00	9.3%	8.9%	0.01
Cerebrovascular Diseases	18.1%	3.2%	0.48	18.1%	17.8	0.01
*Lab Data*						
BMI (kg/m^2^)	28.2 ± 6.1	29.1 ± 6.4	0.14	28.2 ± 6.1	28.2 ± 6.0	0.00
HbA1c	6.4 ± 1.7	6.2 ± 1.6	0.12	6.4 ± 1.7	6.4 ± 1.7	0.00

PSM: Propensity Score Matching; SD: Standard Deviation; SMD: Standardized mean difference; CKD: Chronic Kidney Disease; BMI: Body Mass Index; HbA1c: Hemoglobin A1c; HTN: Hypertension; HLD: Hyperlipidemia, N: Number of patients.

**Table 1c.** Baseline characteristics of RAO and control cohort before and after propensity score matching.

	Before PSM			After PSM		
	RAO (N = 15,372)	Control (N = 3,076,291)	SMD	RAO (N = 15,372)	Control (N = 15,372)	SMD
Age at index, years (Mean +/- SD)	73.3 ± 8.8	68.5 ± 8.7	0.55	73.3 ± 8.8	73.5 ± 8.8	0.02
*Gender*	* *					* *
Female	42.5%	52.6%	0.20	42.5%	42.3%	0.00
Male	54.0%	41.6%	0.25	54.0%	54.0%	0.00
Unknown Gender	3.5%	5.8%	0.11	3.5%	3.7%	0.01
*Race*						
White	71.8%	71.1%	0.02	71.8%	72.6%	0.02
Black	10.9%	10.7%	0.01	10.9%	10.7%	0.01
Asian	2.1%	2.9%	0.05	2.1%	1.9%	0.01
Unknown Race	15.2%	15.3%	0.00	15.2%	14.8%	0.01
*Systemic comorbidities*						
Hypertension (HTN)	72.9%	33.5%	0.79	72.9%	72.9%	0.00
Diabetes Mellitus	33.8%	12.8%	0.50	33.8%	32.9%	0.02
Hyperlipidemia (HLD)	56.1%	22.4%	0.69	56.1%	55.9%	0.00
Ischemic Heart Diseases	35.3%	6.1%	0.72	35.3%	35.5%	0.00
Chronic Kidney Disease (CKD)	19.7%	3.2%	0.52	19.8%	19.3%	0.01
Nicotine Dependence	14.9%	9.3%	0.17	14.9%	14.1%	0.02
Cerebrovascular Diseases	50.9%	3.2%	1.07	50.9%	50.9%	0.00
*Lab Data*						
BMI (kg/m^2^)	29.0 ± 6.4	29.9 ± 7.0	0.13	29.0 ± 6.5	28.7 ± 6.2	0.05
HbA1c	6.3 ± 1.4	6.3 ± 1.8	0.00	6.3 ± 1.4	6.3 ± 1.5	0.00

PSM: Propensity Score Matching; SD: Standard Deviation; SMD: Standardized mean difference; CKD: Chronic Kidney Disease; BMI: Body Mass Index; HbA1c: Hemoglobin A1c; HTN: Hypertension; HLD: Hyperlipidemia, N: Number of patients.

**Figure 2.**
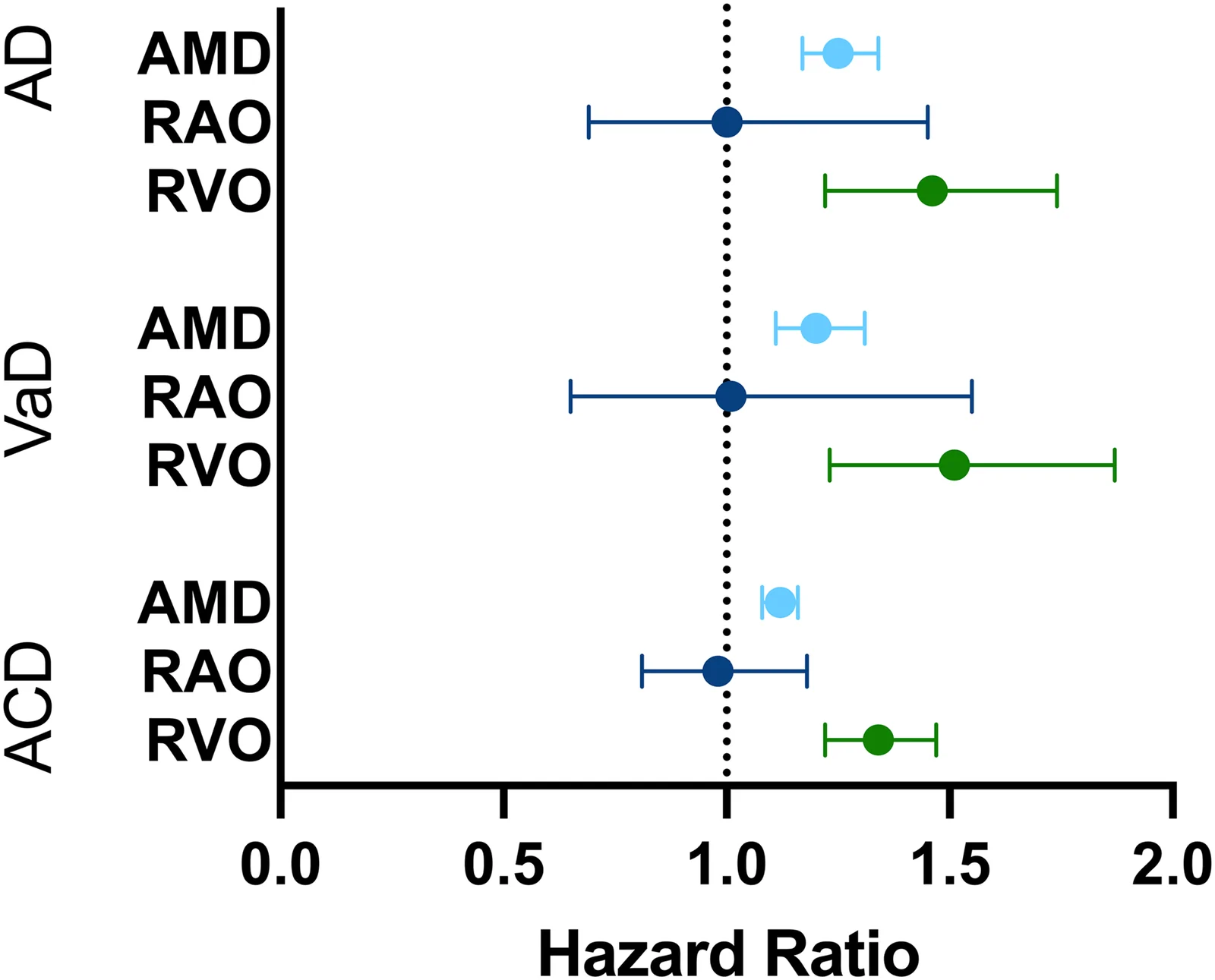
Hazard ratios for the development of dementia pathology in patients with macular degeneration or vascular occlusions. AD: Alzheimer's Disease, VaD: vascular dementia, ACD: all-cause dementia, AMD: age-related macular degeneration, RAO: retinal artery occlusion, RVO: retinal vein occlusion.

Table 2 demonstrates the HR of developing AD, VaD, and ACD in patients with AMD, RAO, and RVO compared to the PC cohort following initial retinal disease diagnosis. The average follow up was 45.0 ± 36.7 months, 40.6 ± 36.8 months, and 46.6 ± 37.9 months in the AMD, RAO, and RVO cohorts, respectively (PC cohort had matched follow up times). AMD was associated with a significantly higher incidence of AD (2.26% vs. 1.50%, HR 1.25, 95% CI 1.17–1.34, *P* < 0.001), VaD, (1.10% vs. 0.85%, HR 1.20, 95% CI 1.11–1.31, *P* < 0.001), and ACD (6.87% vs. 5.32%, HR 1.12, 95% CI 1.08–1.16, *P* < 0.001) when compared to controls (PC). For RAO, the HR was neutral for AD (0.36% for both cohorts, HR 1.00, 95% CI 0.69–1.45, *P* = 1.00), VaD (0.33% vs. 0.30%, HR 1.01, 95% CI 0.65–1.55, *P* = 0.96), and ACD (1.25% vs. 1.32%, HR 0.98, 95% CI 0.81–1.18, *P* = 0.83). In RVO patients, there was a higher risk of developing AD (1.23% vs. 0.98%, HR 1.46, 95% CI 1.22–1.74, *P* < 0.001), VaD (1.03% vs. 0.65%, HR 1.51, 95% CI 1.23–1.87, *P* < 0.001), and ACD (4.55% vs. 3.42%, HR 1.34, 95% CI 1.22–1.47, *P* < 0.001) compared to controls (Figure 2). A sensitivity analysis to identify outcomes of cohorts without prior or subsequent exclusion of comparative retinal diagnoses can be found in Supplemental Table 2.

**Table 2.** Development of Alzheimer's disease, vascular dementia, and all-cause dementia in AMD, RAO, and RVO compared to matched controls following diagnosis.

	Retinal disease cases	Controls		
	Events, n (%)	Events, n (%)	Hazard Ratio (95% CI)	*P* value
*Alzheimer's Disease*				
AMD	2,059 (2.26)	1,373 (1.50)	1.25 (1.17–1.34)	<.001
RAO	55 (0.36)	55 (0.36)	1.00 (0.69–1.45)	1.00
RVO	430 (1.23)	343 (0.98)	1.46 (1.22 –1.74)	<.001
*Vascular Dementia*				
AMD	1,003 (1.10)	770 (0.85)	1.20 (1.11–1.31)	<.001
RAO	51 (0.33)	46 (0.30)	1.01 (0.65–1.55)	.96
RVO	359 (1.03)	226 (0.65)	1.51 (1.23–1.87)	<.001
*All-Cause Dementia*				
AMD	6,270 (6.87)	4,859 (5.32)	1.12 (1.08–1.16)	<.001
RAO	192 (1.25)	203 (1.32)	0.98 (0.81–1.18)	.83
RVO	1587 (4.55)	1192 (3.42)	1.34 (1.22–1.47)	<.001

AMD: Age-related macular degeneration, RAO: Retinal artery occlusion, RVO: Retinal vein occlusion; CI: confidence interval.

**Figure 3.**
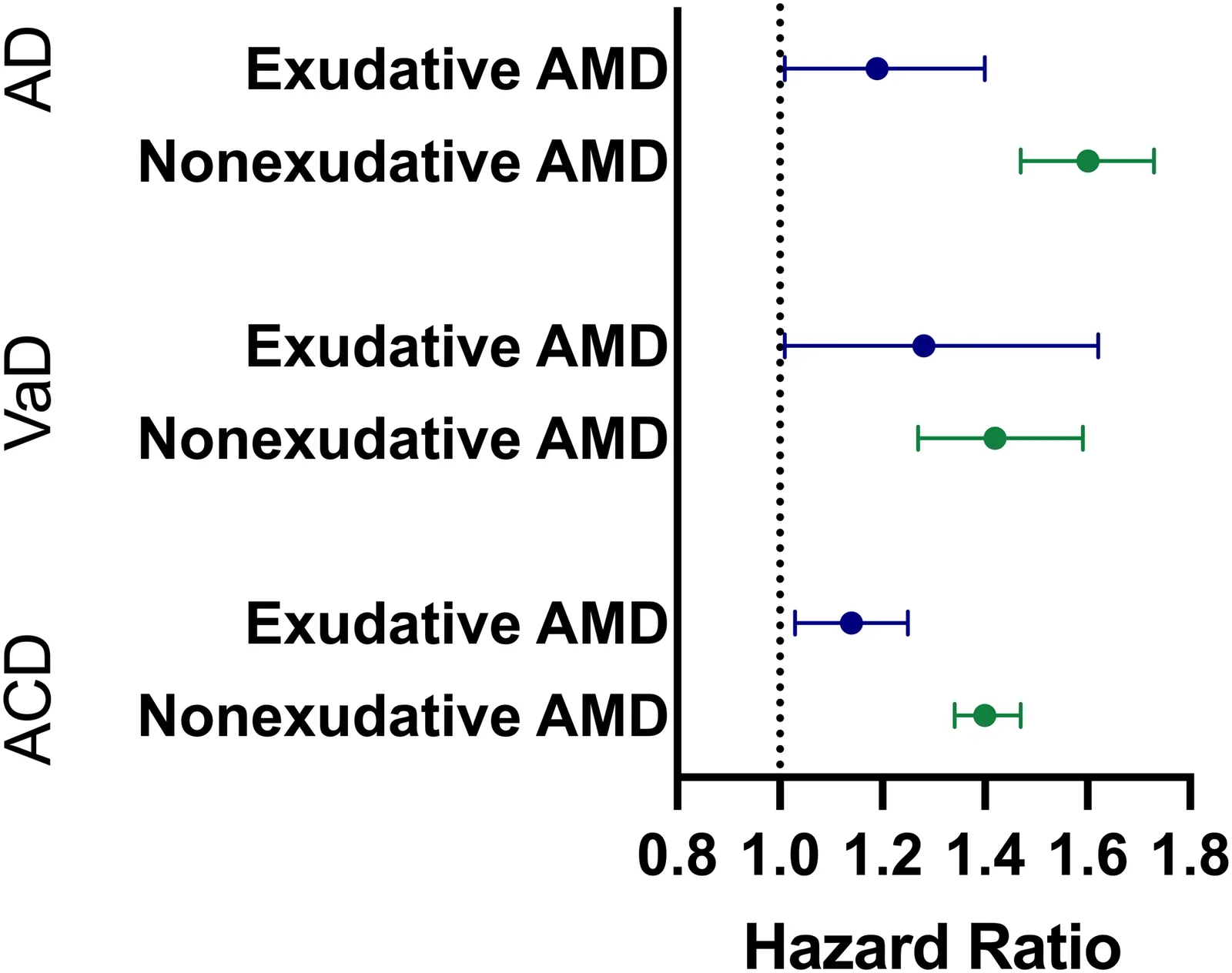
Hazard ratios for the development of dementia pathology in patients with exudative and non-exudative macular generation. AD: Alzheimer's Disease, VaD: vascular dementia, ACD: all cause dementia, AMD: age-related macular degeneration.

Additional analyses in Table 3 evaluated the development of AD, VaD, and ACD among patients with nonexudative and exudative AMD compared to matched controls. Nonexudative AMD was associated with an elevated risk of developing AD (2.77% vs 1.46%; HR, 1.60; 95% CI, 1.47–1.73), VaD (1.31% vs 0.82%; HR, 1.42; 95% CI, 1.27–1.59), and ACD (7.94% vs 5.21%; HR, 1.40; 95% CI, 1.34–1.47) compared to controls. Similarly, exudative AMD was associated with an increased risk of developing AD (0.54% vs 0.29%; HR, 1.19; 95% CI, 1.01–1.40), VaD (0.30% vs 0.13%; HR, 1.28; 95% CI, 1.01–1.62), and ACD (1.64% vs 0.81%; HR, 1.14; 95% CI, 1.03–1.25) when compared to controls (Figure 3).

**Table 3.** Development of AD, VaD, and ACD in patients with nonexudative and exudative AMD versus matched controls following PSM.

	Nonexudative AMD N = 53,603	Control N = 53,603		Exudative AMD N = 14,234	Control N = 14,234	
Dementia Type	Events, n (%)	Events, n (%)	Hazard Ratio (95% CI)	Events, n (%)	Events, n (%)	Hazard Ratio (95% CI)
AD	1,484 (2.77)	783 (1.46)	1.60 (1.47–1.73)	77 (0.54)	41 (0.29)	1.19 (1.01–1.40)
VaD	700 (1.31)	437 (0.82)	1.42 (1.27–1.59)	42 (0.30)	18 (0.13)	1.28 (1.01–1.62)
ACD	4,255 (7.94)	2790 (5.21)	1.40 (1.34–1.47)	234 (1.64)	115 (0.81)	1.14 (1.03–1.25)

AD: Alzheimer's disease; VaD: vascular dementia; ACD: all cause dementia; AMD: Age-related macular degeneration; CI: confidence interval; n: Number of events; N: number of patients.

**Figure 4.**
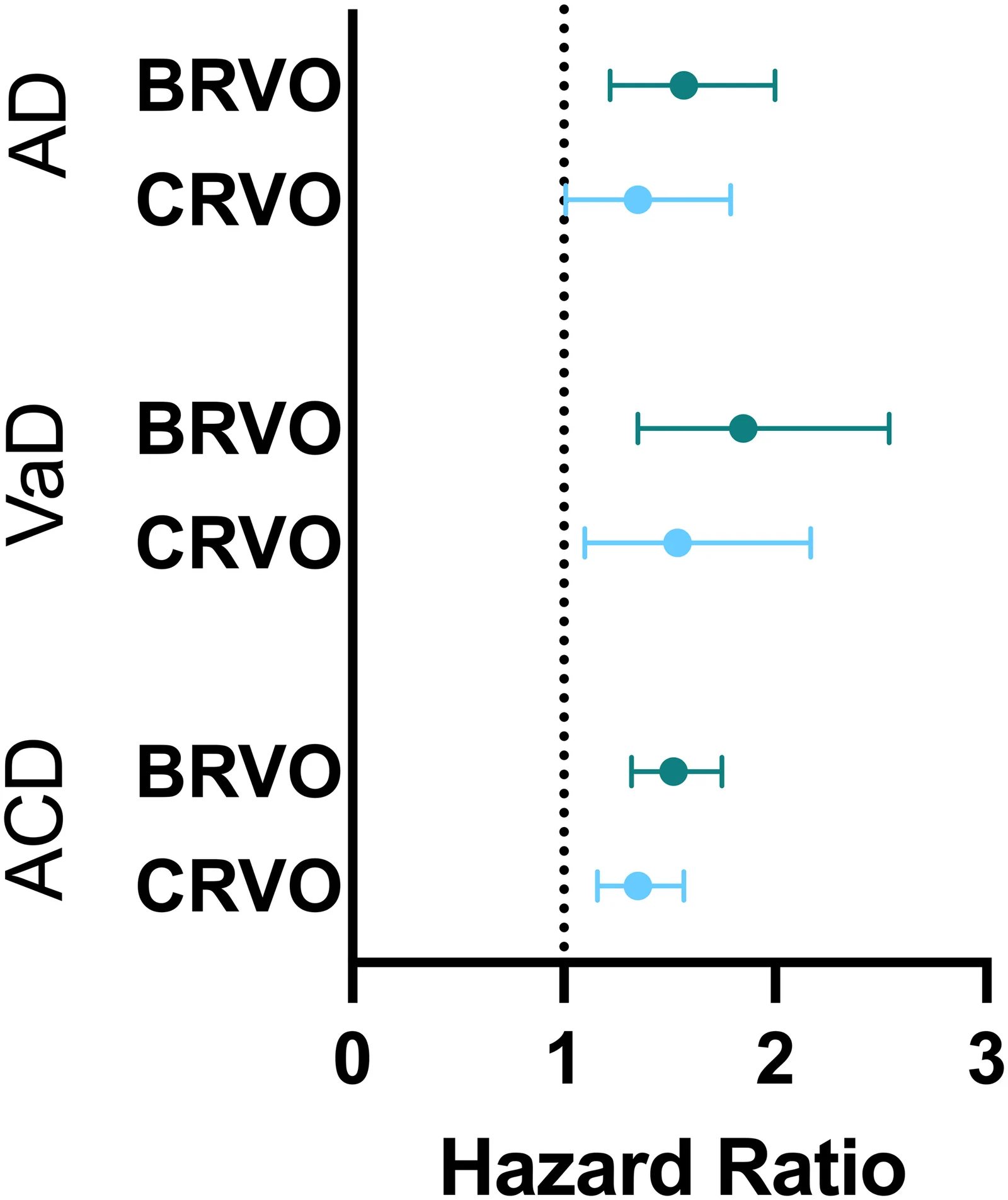
Hazard ratios for the development of dementia pathology in patients with branch retinal vein occlusion and central retinal vein occlusion. AD: Alzheimer's Disease, VaD: vascular dementia, ACD: all cause dementia, BRVO: branch retinal vein occlusion, CRVO: central retinal vein occlusion.

Further analyses in Table 4 assessed the development of AD, VaD, and ACD in patients with CRVO and BRVO compared to matched controls following initial retinal disease diagnosis. CRVO was associated with a higher risk of developing AD (0.36% vs 0.26%; HR, 1.35; 95% CI, 1.01–1.79), VaD (0.35% vs 0.16%; HR, 1.54; 95% CI, 1.10–2.17), and ACD (1.24% vs 0.76%; HR, 1.35; 95% CI, 1.16–1.57) compared to controls. Similarly, BRVO was also associated with an elevated risk of developing AD (1.40% vs 0.75%; HR, 1.57; 95% CI, 1.22–2.00) VaD (0.94% vs 0.47%; HR, 1.85; 95% CI, 1.35–2.54), and ACD (4.22% vs 2.54%; HR, 1.52; 95% CI, 1.32–1.75) compared to controls (Figure 4).

**Table 4.** Development of AD, VaD, and ACD in patients with CRVO and BRVO versus control following PSM.

	CRVO N = 6,964	Control N = 6,964		BRVO N = 11,168	Control N = 11,168	
Dementia Type	Events, n (%)	Events, n (%)	Hazard Ratio (95% CI)	Events, n (%)	Events, n (%)	Hazard Ratio (95% CI)
AD	25 (0.36)	18 (0.26)	1.35 (1.01–1.79)	156 (1.40)	84 (0.75)	1.57 (1.22–2.00)
VaD	24 (0.35)	11 (0.16)	1.54 (1.10–2.17)	105 (0.94)	53 (0.47)	1.85 (1.35–2.54)
ACD	86 (1.24)	53 (0.76)	1.35 (1.16–1.57)	471 (4.22)	284 (2.54)	1.52 (1.32–1.75)

AD: Alzheimer's disease; VaD: vascular dementia; ACD: all cause dementia; BRVO: branch retinal vein occlusion; CRVO: Central retinal vein occlusion; CI: confidence interval; n: Number of events; N: number of patients.

## Discussion

In this retrospective cohort study, individuals diagnosed with AMD or RVO were significantly more likely to develop dementia after adjusting for demographic and systemic comorbidities. RAO was not associated with an increase in dementia risk. Subgroup analysis revealed that both exudative and nonexudative AMD as well as branch and central RVO shared an increased risk for subsequent dementia diagnoses.

AD is a progressive dementia characterized by the accumulation of extracellular amyloid plaques and intracellular neurofibrillary tangles.^18^ Johnson et al identified Aβ peptide, a major constituent of amyloid plaque in AD, in the drusen of AMD patients.^19^ Aβ peptide has since been shown to play a central role in AMD, inducing inflammation and activating complement components.^20,21^ Although drusen is not present in all AMD phenotypes, it is a frequent finding across AMD stages and could potentially explain the observed associations between AMD and AD.^22^ Supporting this, a recent metanalysis by Tsai et al demonstrated a significant albeit modest association between AMD and the development of both AD and ACD (HR: 1.21, 95% CI 1.03–1.43; HR 1.22, 95% CI 1.01–1.47, respectively).^23^ On the contrary, a recent study of the US population using TriNetx showed a decreased likelihood of developing AD in patients with AMD 5 and 7 years after diagnosis. That study notably used a much younger population which may explain the disparate results when compared to the present study, as AMD was no longer protective 10 years after diagnosis.^24^

In contrast to AD, the relationship between AMD and VaD is not well elucidated. A hypothesized mechanism of AMD progression involves Aβ peptide deposition in the choroid, affecting basement membrane attachment and causing vascular damage through complement-mediated injury, leading to choriocapillaris loss and retinal ischemia.^25,26^ This mechanism may occur throughout the brain vasculature, leading to VaD. Using optical coherence tomography angiography (OCTA), Qui et al described correlations between reduced peripapillary capillary density and AMD; similar OCTA findings were found to be predictors of VaD.^27^

Our analysis of RVO revealed strong associations with AD, VaD and ACD. While the pathophysiology of RVO is poorly understood, advanced age, local venous stasis, endothelial damage, and hypercoagulability may play a role.^28^ The significant associations between RVO and dementia may be due to a shared pathophysiology. In a retrospective case-control study by Cho et al, magnetic resonance imaging findings of 125 patients with RVO were found to have a higher prevalence of cerebral small vessel disease compared to an age-matched cohort.^29^ Additionally, multiple studies have shown that cerebral vascular dysfunction is a risk factor for AD and VaD, which may blur the distinction between these dementias.^30–32^ Yet, few studies have investigated a direct link between RVO and incident dementia, with mixed findings. In a recent Danish cohort study, Clausen and colleagues identified RVO as a risk factor for ACD (HR 1.09, 95% CI 1.01–1.18), but the associations with VaD and AD were weak and non-significant.^33^ In our analysis, RVO was also associated with all dementias, but the association between RVO and AD was unexpectedly stronger than AMD and AD, underscoring the hypothesis that vascular dysfunction is implicated in dementia diseases, particularly AD.

In this study, no association was observed between RAO and subsequent dementia diagnoses. A possible explanation for these findings is that RAO is often a sequalae of underlying systemic disease, rather than local vascular or neurologic dysfunction and can be considered a stroke equivalent.^34^ In contrast, RVO and AMD are influenced by a broader array of systemic and intraocular risk factors not fully captured by our analysis, including ocular HTN and prior intraocular surgery.^7^ Thus, adjusting for covariates, particularly CVD, HTN, HLD, and DM, may have attenuated the observed risk in the RAO cohort more strongly than in the RVO or AMD cohorts.

This study also employed subgroup analysis of AMD. While it would be expected that advanced AMD would correlate to increased AD incidence, this study found that nonexudative AMD had a higher correlation than exudative AMD with both AD and ACD. Similarly, a meta-analysis by Tsai et al examining almost 8 million patients found that patients with nonexudative AMD had an increased risk of developing ACD, while no such association was found for exudative AMD.^35^ While the mechanism is not yet known, a growing body of evidence has linked angiogenesis with neurodegenerative disease, finding increased levels of vascular endothelial growth factor (VEGF) in the plasma and cerebrospinal fluid of patients with AD.^36–39^ Given this association, one author has proposed that intravitreal anti-VEGF treatment given for exudative AMD may exert a protective effect, citing a small but nonsignificant reduction in the development of AD in patients with exudative AMD receiving intravitreal anti-VEGF injections.^23^ However, other studies have criticized this theory on the basis of poor systemic absorption of intravitreal injections.^24^

This was also the first study to incorporate subgroup analysis for RVO. Both BRVO and CRVO were associated with an increased risk for all dementias. Interestingly, BRVO held stronger associations with all subsequent dementias compared to CRVO. This may also be partially mediated by the increased rate of intravitreal anti-VEGF treatment in patients with CRVO. Another possibility may be alternate pathways of pathophysiology given BRVO and CRVO have differential risk factors; while BRVO is more closely linked to AV nicking, high BMI, and local compression, CRVO is more strongly associated with systemic factors such as DM and thrombophilias.^7^

Dementia affects approximately 6 million individuals over age 60 in the U.S.^40^ While screening is not currently recommended, early identification can allow physicians to optimize and treat dementia risk factors and provide individuals and families the time to make informed choices about their future. The results of this study provide a unique opportunity in this regard, as ophthalmic imaging is easier to obtain than many other screening exams and is often needed for other purposes. One study demonstrated that deep learning algorithms have excellent performance detecting early onset AD and mild cognitive impairment by analyzing optical coherence tomography images.^41^

A key strength of our study is its large national cohort design along with extensive PSM for multiple known risk factors that provided a robust sample with high statistical power. This study also examines a U.S. cohort, which may have differential risk factors compared to other studies.^42^ Additionally, subgroup analysis containing a large sample size was employed, revealing new associations. To the authors’ knowledge, this is the largest single investigation examining multiple retinal diseases and dementia subtypes.

This study also had several limitations. First, data accuracy warrants consideration. Because TriNetX aggregates data from multiple healthcare organizations, the validity and consistency of ICD-10 coding cannot be independently verified, and detailed clinical information—such as disease severity—is limited. This may explain the higher than anticipated prevalence of RAO compared to previous population level analyses.^43,44^ Additionally, the retrospective design introduces the possibility of selection bias. Differences in healthcare utilization may also exist, as patients with retinal disease often undergo more frequent follow-up evaluations, which may increase opportunities for earlier detection of dementia and cognitive decline. Also, the use of TriNetX analytics tools may introduce analytical limitations. Although PSM was utilized to reduce the effect of cofounders, this analysis cannot eliminate unmeasured covariates such as family history of AD, visual acuity, genetics, and socioeconomic or lifestyle factors.

Lack of visual acuity is a particular limitation of this study as it is a known risk factor for dementia.^45,46^ Because we were unable to adjust for visual acuity, we are unable to definitively comment on whether low vision is a substantial mediator of the results found in this study. However, the results of this analysis are unlikely to be solely explained by severe vision loss. In our study, non-exudative AMD had a stronger association with dementia than exudative AMD, which often leads to more severe visual impairment. Similarly, RVO was associated with dementia, whereas RAO, which usually causes more profound acute vision loss, was not.

Furthermore, balanced covariates may not be fully captured in our analysis, as many disease processes exist on a spectrum and are imperfectly categorized by ICD codes. This can be seen when using diagnosis codes such as nicotine dependence as proxies for important covariates (i.e., smoking status). Lastly, some subgroup analyses reached only borderline statistical significance. Considering our study involved multiple tests for significance, the likelihood of a type 1 error is increased. Therefore, these findings should be interpreted conservatively.

Overall, this nationwide population-based study suggests that retinal disease may be correlated with an increased risk of dementia development following retinal disease diagnosis in patients 65 years or older. Specifically, AMD and RVO may hold associations with AD, VaD and ACD. RAO did not increase risk for any dementia diagnosis. Further studies are necessary to correlate the underlying pathophysiology and causation of these diseases.

## Supplemental Material

sj-docx-1-ejo-10.1177_11206721261438587 - Supplemental material for Analyzing the association between age-related macular degeneration, retinal vascular occlusions, and dementia Supplemental material, sj-docx-1-ejo-10.1177_11206721261438587 for Analyzing the association between age-related macular degeneration, retinal vascular occlusions, and dementia by Ahmed Alshaikhsalama, Krista Thompson, Haafiz Hashim and Kishan G Patel in European Journal of Ophthalmology

sj-docx-2-ejo-10.1177_11206721261438587 - Supplemental material for Analyzing the association between age-related macular degeneration, retinal vascular occlusions, and dementia Supplemental material, sj-docx-2-ejo-10.1177_11206721261438587 for Analyzing the association between age-related macular degeneration, retinal vascular occlusions, and dementia by Ahmed Alshaikhsalama, Krista Thompson, Haafiz Hashim and Kishan G Patel in European Journal of Ophthalmology
