# Supplementary material for: Analyzing the association between age-related macular degeneration, retinal vascular occlusions, and dementia
Source: Eur J Ophthalmol. 2026 Apr 7;36(5):1343–53. doi: 10.1177/11206721261438587 (PMC13490658; doi:10.1177/11206721261438587)
Supplement: Supplementary file 1 [file sj-docx-1-ejo-10.1177_11206721261438587.docx]

| **Diagnosis** | **ICD-10** |
| --- | --- |
| **Retinal Disease** |  |
| Age-related macular degeneration | H35.31 (nonexudative), H35.32 (exudative) |
| Retinal artery occlusion | H34.1 (central), H34.23 (branch) |
| Retinal vein occlusion | H34.81 (central), H34.83 (branch) |
| **Dementia** |  |
| Alzheimer’s disease | F00, G30 |
| Vascular dementia | F01 |
| All-cause dementia | F00-F03, G30 |
| **Comorbidities** |  |
| Hypertension | I10 |
| Diabetes mellitus | E08-E13 |
| Hyperlipidemia | E78.5 |
| Ischemic heart disease | I25 |
| Chronic kidney disease | N18 |
| Nicotine dependence | F17 |
| Cerebrovascular disease | I63 |

eTable1. List of ICD-10 codes used in this study.
