# Supplementary material for: Analyzing the association between age-related macular degeneration, retinal vascular occlusions, and dementia
Source: Eur J Ophthalmol. 2026 Apr 7;36(5):1343–53. doi: 10.1177/11206721261438587 (PMC13490658; doi:10.1177/11206721261438587)
Supplement: Supplementary file 2 [file sj-docx-2-ejo-10.1177_11206721261438587.docx]

**Supplemental Table 2.** Development of Alzheimer’s Disease, Vascular Dementia, and All-Cause Dementia in AMD, RAO, and RVO using baseline classification without prior or subsequent exclusion compared to matched controls.

|  | **Retinal Disease Cases** | **Controls** |  |  |
| --- | --- | --- | --- | --- |
|  | **Events, n (%)** | **Events, n (%)** | **Hazard Ratio**  **(95% CI)** | P value |
| ***Alzheimer's Disease*** |  | | |  |
| AMD | 2,158 (2.23) | 1,500 (1.55) | 1.25 (1.16–1.35) | <.001 |
| RAO | 402 (1.88) | 267 (1.25) | 1.48 (1.17–1.87) | .001 |
| RVO | 834 (1.84) | 557 (1.23) | 1.33 (1.13–1.56) | <.001 |
| ***Vascular Dementia*** |  | | |  |
| AMD | 1,113 (1.15) | 832 (0.86) | 1.17 (1.05–1.30) | .004 |
| RAO | 331 (1.55) | 237 (1.11) | 1.40 (0.81–2.00) | .14 |
| RVO | 607 (1.34) | 376 (0.83) | 1.44 (1.19–1.74) | <.001 |
| ***All-Cause Dementia*** |  | | |  |
| AMD | 6,629 (6.85) | 5,371 (5.55) | 1.08 (1.04–1.13) | <.001 |
| RAO | 1,421 (6.65) | 1,066 (4.99) | 1.33 (0.99–1.50) | .053 |
| RVO | 2,810 (6.20) | 1,985 (4.38) | 1.28 (1.18–1.40) | <.001 |

Abbreviations: AMD: Age-related macular degeneration, RAO: Retinal artery occlusion, RVO: Retinal vein occlusion; CI: confidence interval.
